# Microfluidic Based Physical Approaches towards Single-Cell Intracellular Delivery and Analysis

**DOI:** 10.3390/mi12060631

**Published:** 2021-05-28

**Authors:** Kiran Kaladharan, Ashish Kumar, Pallavi Gupta, Kavitha Illath, Tuhin Subhra Santra, Fan-Gang Tseng

**Affiliations:** 1 Department of Engineering and System Science, National Tsing Hua University, Hsinchu 300044, Taiwan; kiran.911989@gmail.com (K.K.); aashu9t@gapp.nthu.edu.tw (A.K.); 2 Department of Engineering Design, Indian Institute of Technology Madras, Chennai 600036, India; pgupta1304@gmail.com (P.G.); kavithaillath24@gmail.com (K.I.)

**Keywords:** physical methods for intracellular delivery, single-cell, microfluidic devices, transfection efficiency, cell viability

## Abstract

The ability to deliver foreign molecules into a single living cell with high transfection efficiency and high cell viability is of great interest in cell biology for applications in therapeutic development, diagnostics, and drug delivery towards personalized medicine. Various physical delivery methods have long demonstrated the ability to deliver cargo molecules directly to the cytoplasm or nucleus and the mechanisms underlying most of the approaches have been extensively investigated. However, most of these techniques are bulk approaches that are cell-specific and have low throughput delivery. In comparison to bulk measurements, single-cell measurement technologies can provide a better understanding of the interactions among molecules, organelles, cells, and the microenvironment, which can aid in the development of therapeutics and diagnostic tools. To elucidate distinct responses during cell genetic modification, methods to achieve transfection at the single-cell level are of great interest. In recent years, single-cell technologies have become increasingly robust and accessible, although limitations exist. This review article aims to cover various microfluidic-based physical methods for single-cell intracellular delivery such as electroporation, mechanoporation, microinjection, sonoporation, optoporation, magnetoporation, and thermoporation and their analysis. The mechanisms of various physical methods, their applications, limitations, and prospects are also elaborated.

## 1. Introduction

The cell is the most fundamental independent unit of life which transmits information through molecules. Delivering molecules and other external materials into the cells is a major step in understanding cell functions and reprogramming cell behavior. Therefore, intracellular delivery is extremely important for understanding biological systems and treating diseases. Introducing small molecules, exogenous molecules, and macromolecules such as DNA, drug molecules, enzymes, peptides, and antibodies into cells is a huge challenge to achieve their full potential for a range of research and therapeutic applications [1,2,3]. Researchers have been conducting experiments for decades for developing and adapting molecules into prospective cargo to be delivered into the intracellular environment [4]. The majority of these cargos are membrane-impermeable and therefore need physical energy for intracellular delivery [5]. Cargos for the delivery are varied in different aspects such as size, shape, architecture, and chemical properties. The range of molecules varies from small hydrophilic molecules [6] of 1 nm to large micron-sized organelles and microorganisms which can be as large as the size of the cell itself. Thus, the primary challenge in all these cargos that they are cell impermeable and must be delivered into the intracellular environment without much damage to the cell.

Intracellular delivery approaches are broadly divided into two types: (i) By using biological or viral vectors technique and (ii) by using physical techniques (non-viral chemical vectors) to deliver molecules into cell cytoplasm or nucleus. Viral vectors are the most favored method for in vivo gene transfer as they are the most clinically advanced delivery agents. They have been used for most of the clinical and pre-clinical trials [7]. They have an excellent gene delivery efficiency and have the capability to introduce transduction of target cells and tissues permanently [8]. Recent developments of viral vector platforms have been based on adenovirus, retrovirus, lentivirus, and various other viruses. While viral vector delivery is highly effective in their application of the insertion of therapeutic genes into the target cell, it may have some adverse consequences too [9]. The prominent disadvantages of the viral vectors are the following: (1) they are toxic and can cause inflammatory immune responses [10], (2) safety issues, (3) risk of insertional genotoxicity, (4) labor-intensive and costly protocols, (5) lower packaging capacity, and (6) risk of genotoxicity [11]. These limitations concerning viral vectors have inspired the development of non-viral vectors [12,13].

For the treatment of diseases in the intracellular microenvironment, numerous tools have been developed that could modify the genes, including nucleic acids (such as DNA plasmids, messenger RNA (mRNA), microRNA (miRNA), and small interfering RNA (siRNA). The nucleic acid can overexpress certain genes in cells for various functions, inhibitor proteins as drugs for anti-tumor treatment, molecular beacon probes, and DNA origami enabling investigation of intracellular environment and bio-sensors and nano-devices for manipulation at the molecular level [14,15,16].

For the intracellular delivery of proteins, new developments in nanocarriers have been investigated [17,18], but these techniques are still in the development stage and the evolution of next-generation nanocarriers will be driven by intracellular delivery of genome editing complexes [19,20]. Plenty of research reveals that the majority of carriers enter cells through endocytosis and then escapes into the cytoplasm [21,22].

Without causing any immune response as viral vectors, chemical vectors deliver some of the benefits as the former. However, the major limitations of chemical vectors are low efficiency and inadequate delivery, especially in the case of primary cells and stem cells. Chemical vectors have to cross all biological barriers for cargo delivery into the nucleus, while viral vectors inherently have the ability of nuclear import. Despite the limitations, numerous aspects of this technique were discussed in various reviews, where cationic polymers and cationic lipids as non-viral vectors for gene transfection are established in detail [23,24,25]. Different strategies have been investigated by scientists to enhance the chemical vector-based cargo delivery and these investigations focus on modifying cell-penetrating peptides or proteins (CPP) [26] or endosomal escape to transfect cargo molecules into the cytoplasm directly [25]. The major challenges for chemical vectors lie in achieving high cell viability and difficult-to-transfect molecules in both in vitro as well as in vivo.

During the past decade, various microfluidic-based physical methods for single-cell intracellular delivery have been gaining importance and major advancements have been happening in this area of transfection. However, viral and non-viral chemical approaches are still the more accepted techniques in clinical and laboratory research applications [27,28]; intracellular delivery, especially, did not start to emerge until the 1980s [29]. Physical methods are capable to overcome some of the major limitations of chemical and vector-based intracellular delivery techniques [30,31]. Moreover, viral and non-viral approaches are considered as bulk delivery techniques where millions of cells are transfected together.

The study of human disease mechanisms is not easy because of the variation in gene expression and cell physiology in a selected population. Single-cell measurement mechanisms can give precise knowledge of the cellular and molecular interactions compared to bulk cell measurements and can help in developing diagnostic and therapeutic tools [32,33,34,35].

In the last two decades due to the rapid development of micro/nanotechnologies with the integration of chemical, biomedical, chemistry, and life science, the new era began and is known by its micro/nanofluidic devices. These are not only useful for cell manipulation, separation, therapy, and lysis but it can also perform cellular mechanical, electrical, and optical characterization with minimum consumption of power and sample amount [36,37,38,39,40,41].

Physical method based reviews have been published previously for cellular delivery and analysis. However, most of the individual methods such as microinjection, electroporation, mechanoporation, sonoporation, and photoporation demonstrated bulk analysis of millions of cells together except microinjection and electroporation. However, the difference in this review is that, we have comprehensively discussed different microfluidic-based physical approaches towards single-cell intracellular delivery and analysis. The relevance and applicability of single-cell approaches have been discussed in the following section. We have broadly classified physical delivery techniques into two sections [42,43]. Firstly, direct penetration methods [44], where cargos are inserted directly into the cell nucleus or cytoplasm. Secondly, methods where disruption of cell membrane occurs due to the application of a physical energy (electrical [45], light [46], magnetic [47], mechanical [48], ultrasonic [49], or thermal [50]).

We broadly emphasize the working principles of each method, device fabrication, and application techniques in different in vitro and in vivo platforms. Lastly, we critically assess each delivery method comparing their performance parameters such as (i) cell viability and transfection efficiency with different cell types and cargo molecules, (ii) effectiveness of the single-cell approach, (iii) possibility to integrate with current workflows, and (iv) adaptability to changeover between delivery of different cell types, sizes, and morphologies.

## 2. Direct Penetration Methods

The direct penetration method overcomes all the resistance to transfect the molecules within the cell or nucleus. In this method, every cargo molecule is delivered as carrier liquid (e.g., microinjection) or by a particle (e.g., particle bombardment). However, for field-induced membrane disruption methods (e.g., electroporation, optoporation, mechanoporation, sonoporation, magnetoporation, and thermoporation), the cell membrane is disrupted systematically and creating transient hydrophilic nano-pores to deliver biomolecules into cells.

### 2.1. Microinjection

The most straightforward physical delivery method is the injection of molecules directly into the cell. Microinjection is considered the most efficient method of direct cargo delivery [51,52]. Conventionally, a macromolecular solution is pressurized using a glass micropipette within a single cell to a particular location [53,54,55]. For extraction and transfer of cellular components between cells, single-cell microinjection is a very effective method for delivering exogenous material into cells [56]. Theoretically, the injected substance is the only independent variable and the variation in it causes drastic changes. Unlike in electroporation (discussed in the later section) where the parameters are difficult to control, microinjection also transfers molecules into the nucleus or cytosol with precision, which is difficult to control in many cases with electroporation. Injection of the cDNAs into cells develops less stress on cells, unlike viral infection and chemical transfection. Thus, the background of cell death is found to be reduced with viral infection or liposome mediated transfections. Furthermore, in the microinjection process, more than one cargo molecule can be injected into different groups of cells in one culture dish, where the un-injected cells serve as built-in controls; this makes the results more compatible. Theoretically, the transfection efficiency of the cells that survived injection is nearly 100%. Any microinjection system will typically comprise of three basic functions [51]: the imaging of the target cell and the micropipette, the positioning of the micropipette, and control of the pressure inside the micropipette. Gueroussov et al. [57] developed a single-cell microinjection technique to study the delivery of mRNA in cells. In this method, mRNA and cloned plasmid DNA of 200 and 50–200 μg/mL concentration were injected with Oregon Green 488 (OG)-conjugated 70 kDa dextran (1 mg/mL) into COS-7 cells (green monkey kidney cell). The microinjection method proves to be better for the study of mRNA transport between the subcellular components compared to other techniques of transfection because of the speedy mRNA export. However, cellular level variability in mRNA quantification could result from variations in the flow rate of the needle. Taverna et al. [58] tried to disrupt the plasma membrane of a single neuronal progenitor cell in an organotypic tissue slice with the help of microinjection. The balance of apical progenitor proliferation versus differentiation was drastically changed upon microinjection of isolated Polyadenylated RNA of Proliferating mouse neural stem cell line (NS-5 prol poly-A+ RNA). The state of a single cell in a tissue can be utilized to observe developmental aspects and the function of different proteins in cellular differentiation. Zhang e al. [56] performed microinjections in 11 days on primary fetal cortical cultured neurons with Eppendorf Microinjector 5246 using Burleigh Micromanipulator MIS-5000. The microneedle was made of thin-walled glass capillaries using a Flaming/Brown micropipette puller, where the diameter of the tip was about 0.5 μm taken from 1.0 mm outer diameter and 0.5 mm inner diameter. An injection volume of 25 pL was generated for an injection time of 0.1 s, by the injection pressure of 100 hPa and the compensation pressure of 50 hPa. For an experiment, 200 neurons per trial were utilized and three independent trials were performed by the same method. The injected cells are primary human neurons, with DTR (Dye Terminal Remover) marker dye, which are identified under the fluorescence microscope. Feramisco et al. [59] developed the microinjection method to display the oncogenic property of the oncogenic human ras protein. During this experiment, 35–40 individual cells were injected with a 100–800 μg/mL concentration of protein. Those were studied for the proliferation capability of quiescent cells. The proliferation capability was not achieved in un-injected cells or proto-oncogenic protein microinjected cells while injection of oncogenic protein led to proliferation.

Masani et al. [60] tried to generate non-chimeric transgenic callus using PEG(Polyethylene glycol)-mediated transformation and DNA microinjection from the protoplast of oil palm plants. The microinjection was performed with the help of a Leica DM LFS upright microscope. The major advantage of microinjection application includes increased efficiency, unlike Agrobacterium mediated transformations and particle bombardment.

**Figure 1:**
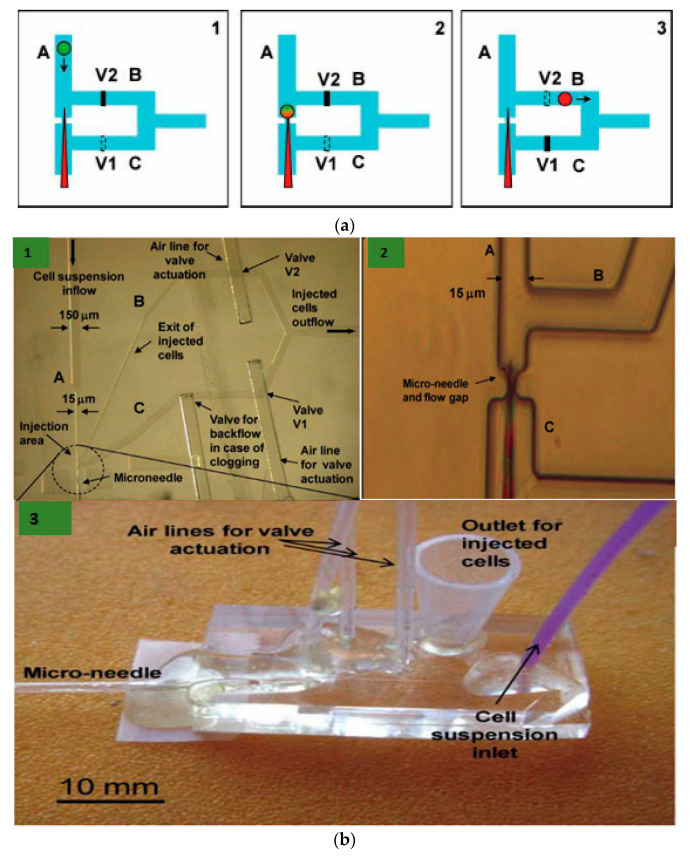
(**a**) Schematic diagram of the microfluidic device based on microinjection system at the single-cell level. (**1**) The fluid stream transports the cell to be injected to a microneedle and valve 1 (V1) is open while valve 2 (V2) is closed. (**2**) The needle helps in piercing the cell and thus performs microinjection. (**3**) Now, V1 is closed and V2 is open. This causes the fluid stream and the volume displaced by the valve V1 process to take the cell off the needle and transport it through channel B into a reservoir. (**b**) (**1**) Microscopic image of the microinjection chip with channels A, B, and C; and valves V1 and V2. (**2**) The zoom-in image of the microinjection area where the cell is impinged by the needle. (**3**) Image of the developed microfluidic platform. Reprinted with permission from ref. [61]. Copyright © 2001 Royal Society of Chemistry.

Afterwards, Adamo and Jensen [61] developed a microfluidic platform for testing single-cell microinjection; this was based on the concept where fluid streams carry the cells to move onto a fixed needle. Contrary to the traditional microinjection approach, where the immobilized cells are injected with microinjection, in the current microfluidic platform the moving cells are guided onto a stationary microneedle for single-cell microinjection. Briefly, the device working mechanism is depicted in Figure 1 and a supply reservoir sends a suspended cell to place firstly in the microchannel (channel A). While channel B is closed, the cell is carried by the stream of fluid in channel C to the needle. (Figure 1(a1)). There are two pneumatic valves V1 and V2, which act as actuation valves that block Channels B and C, respectively. This is performed by deforming a thin elastomeric membrane by the application of pressurized air. The injection is performed when the needle pierces into the cell (Figure 1(a2)). Then, Channel C is closed and channel B is opened (Figure 1(a3)). The cell is lifted off the needle because of the backflow and back pressure created by actuation valve V1 and the injected cells are carried to a collection reservoir. The next cell has to be made ready for injection and the system has to be reset to the state of frame 1. Upon valve activation, there is not much drastic change in flow rate as the pressure drops across channels B and C are made to be equal.

**Figure 2:**
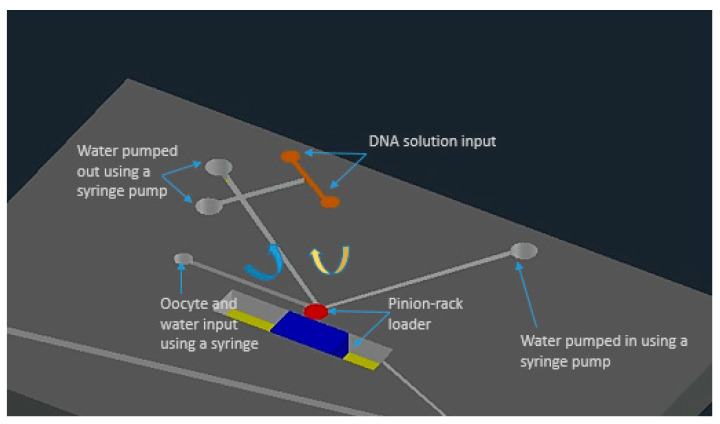
The schematic of the microfluidic chip for microinjection with built-in micro-channels and controlled water flow using syringe pumps. Figure has been redrawn from ref. [62].

K. T. Uning et al. [62] demonstrated an innovative microfluidic microinjection chip comprised of a pinion-rack cell loading mechanism and a 3D bubble injector probe. The cell is placed in the chip and transported to the loader with the help of water flow powered by a syringe pumping in and out from the chip. The cells are separated from one another using the pinion’s gaps and the pinion gear is rotated to transfer the cells individually in front of the bubble injector (Figure 2). The loader is capable of working in a harsh environment and also maintains a high throughput of around 16 gel beads (similar to cells) every minute. This device does not require any special operating skills and therefore permits anyone to perform cell injections without any training.

**Figure 3:**
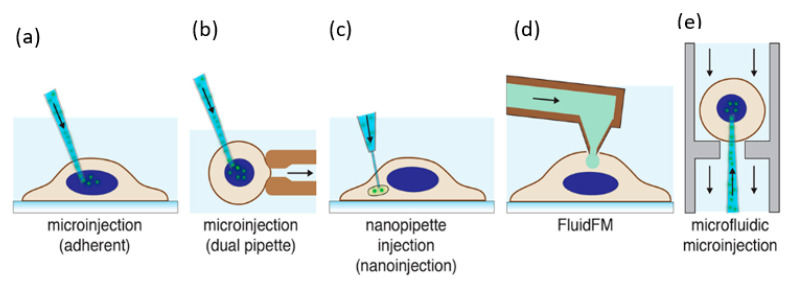
Microinjection for cell transfection. (**a**) Microinjection of an adherent cell using a glass micropipette [63]. (**b**) Microinjection of a suspended cell by a secondary holding pipette [67]. (**c**) Nanopipette injection of an intracellular organelle using a nanotube [68]. (**d**) Injection of cells using a hollow AFM cantilever (FluidFM) [66]. (**e**) Microfluidic microinjection in which a cell is pushed onto a sharp micropipette [61]. Reprinted with permission from ref. [43]. Copyright © 2018 American Chemical Society.

Thus, microinjection is proved to be a flexible delivery technique, capable of transfecting any cargo to the majority of the cell types. It is usually performed with different microinjection systems (given in Figure 3a) connected with glass micropipettes having tip diameters from 0.3 µm to 1.0 µm. The type of cells also determines the performance of microinjection delivery. Small cells like blood cells (<10 µm diameter) show difficulty in microinjection owing to their low tolerance for needle injection. As discussed above, an additional holding pipette is used to keep cells in place in the case of non-adherent cells (Figure 3b) [63]. However, this procedure is complex and time-consuming. In advanced technique, an ultra-fine tip of a diameter of about 100 nm was used to inject single chloroplasts in plant cells (Figure 3c) [64]. An interesting adaptation of the microinjection technique is a technology called FluidFM, which is based on the concept of atomic force microscope (AFM) [65]. It was firstly showcased with the help of hollow cantilevers that, force controlled injection of soluble materials into cells is possible [66] (Figure 3d). Furthermore, microfluidic systems have been adopted to enhance the throughput of microinjection. Adamo et.al. proposed a microfluidic version of microinjection where cells were drawn into a 0.5 µm diameter hollow-tip glass needle embedded in a Polydimethylsiloxane (PDMS) device (Figure 3e) [61].

### 2.2. Particle Bombardment

Unlike needle and jet injections using a carrier fluid, particle mediated delivery transports macromolecules into target cells on a particle carrier. Particle bombardment was primarily utilized to transfect nucleic acids into intact plant cells [69]. Biolistic intracellular delivery has also been called the biolistic process, ballistic particle delivery, micro-projectile bombardment, and, in certain embodiments, the “gene gun” [70]. However, the microfluidic-based particle bombardment has not been developed much, common approaches are discussed in this section.

The biolistic delivery method has been effective in inserting DNA into typically difficult-to-transform plant cells and tissues. However, it is refined for application on smaller cells such as mammalian cells [71]. Briefly, (1) heavy metal particles (usually gold or tungsten, diameter 1–1.5 µm) are used to coat with the cargo molecule and placed in solution on the face of a projectile, (2) a gas shock (e.g., from a chemical explosion, high-voltage (HV) electric spark, or helium discharge) is used to drastically accelerate the projectiles, and (3) the microparticles are released at high speeds into the target cells by stopping the projectile suddenly (e.g., by a mesh) [72,73,74].

Particle bombardment remains to be the predominantly used method for plant transformation. However, it has been accepted as an efficient technique of in vitro and in vivo nucleic acid transfection into mammalian cells and tissues, specifically for gene therapy and genetic immunization applications, where the skin is usually the target tissue [75]. The shallow depth of penetration renders particle bombardment most efficient for single-cell membranes in vitro or superficial tissues (e.g., skin and mucosa) in vivo due to the shallow depth of penetration [75,76]. In addition to these, in vivo gene transfer into liver and muscle tissues has also been showcased.

The biolistic method can be used to transfect DNA to the nucleus directly such as the microinjection method. Due to the limited control over the penetration velocity and distribution of microparticles, the range of results is not precise and predictable. Cells without any successful delivery of microparticles would not display any effect, while higher penetration results in drastic damage to the cells [72]. Various attempts have been made to enhance the accuracy of traditional designs of gene guns along with deeper tissue penetration. In particular, a Bio-Rad hand-held gene gun with an accelerator channel was designed by O’Brien et al. [77] to create a highly focused shot of gold particles at a much lower gas pressure. Another advanced gene gun was proposed by Dileo et al. [78], which uses suspended DNA-coated gold beads as the carrier. Despite being interesting, these enhancements are not sufficient enough to provide groundbreaking changes in applications and operations; they are incapable in influencing wider adoption. O’Brien et al. [79] used 40 nm diameter nanoparticles as projectiles for the biolistic transfections as an alternative to microparticles. The use of nanoparticles enhanced the transfection efficiencies and reduced the issue of tissue damage due to the transfection.

Therefore, the area of penetrating projectiles is mending towards smaller and less destructive projectiles and trying to enhance the uniformity of cell treatment. If the disadvantages mentioned above are successfully addressed, the delivery by penetrating projectile delivery possesses huge potential to deliver evolutionary results.

## 3. Physical Energy Based Membrane Disruption and Intracellular Delivery

In terms of intracellular delivery, the cell membrane is disrupted due to the application of external energy on the cell, which deforms the plasma membrane and helps to create transient hydrophilic membrane pores and deliver various cargos from outside to the cell cytoplasm or nucleus. The primary advantages of field-induced membrane disruption methods are (1) they are capable of delivering cargos of different sizes and shapes and (2) delivery of large cargos are feasible as the majority of the cells can recover from membrane poration of micro-sizes [43]. In the following sub-sections, we will describe different physical methods of single-cell intracellular delivery due to the application of forces in addition to energy, such as mechanical, electrical, optical, thermal, ultrasonic, and magnetic forces disrupting the cell membrane.

### 3.1. Mechanoporation

Mechanoporation is a method of creating transient membrane pores because of the application of mechanical forces, which assists in the delivery of cargo (drugs, plasmids, or molecules) into the cells [80]. These mechanical forces could be shear or compressive stresses acting on the cell membrane when cells are passing through restricted flow conditions [48]. There are mainly three mechanisms through which membrane permeabilization can be performed: (1) solid contact of foreign objects with cells (direct penetration methods), (2) fluid shear forces, and (3) changes in osmotic or hydrostatic pressure.

The microfluidic or lab-on-chip methods can be employed to induce plasma membrane permeability and it assists in the precise control of membrane disruption mechanism. One of the most promising methods is to form transient membrane pores by the mechanical squeezing of a cell as it passes through a microfluidic constriction (Sharei et al. 2013) [80]. The primary purpose of this technique was to deliver almost any biomolecule to any cell type, at high throughput. Different improvisations of the constriction-based delivery methods have been proposed to improve cell viability and efficiency as discussed in the next sections. Pak et al. [81] suggested another intracellular delivery method by gating trans membrane mechanosensitive channels via fluid shear stress. This approach could help to design microfluidic flow channels for shear stress-induced intracellular delivery.

#### 3.1.1. Constriction Channel Based Intracellular Delivery

**Figure 4:**
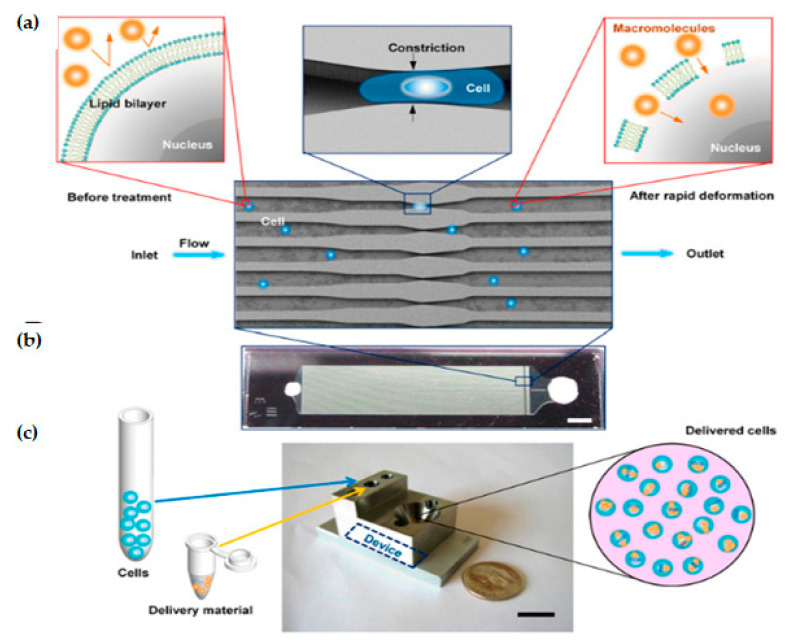
Device design and mechanism. (**a**) Schematic of delivery hypothesis where cell passes across a microfluidic constriction, deforms, and creates transient membrane holes. (**b**) Image of a device consisting of Pyrex bound to silicon for sealing (Scale bar: 2 mm). (**c**) Schematic of the delivery procedure in which cells and delivery material are mixed in the inlet reservoir, run through the chip, and collected in the outlet reservoir (Scale bar: 10 mm). Reprinted with permission from ref. [80]. Copyright © 2013 Proceedings of the National Academy of Sciences.

Sharei et al. presented an innovative microfluidic device [80] where cells are mechanically disrupted and transfect cargos as the cells move through a constriction channel, which is smaller than the cell diameter. The device design and working mechanism are shown in Figure 4.

A single device is comprised of 45 parallel microchannels that are identical, where each microchannel contains one or more constrictions. The length of a single constriction varies from 10–20 μm and the width varies from 4–8 μm. One of the primary reasons to choose a parallel channel system is to improve the cell throughput. It also ensures consistent treatment of cells as any defects or clogging in one channel cannot reduce the flow velocity in neighboring channels. The proposed design has an average throughput rate of 20,000 cells/s and is capable of handling around one million cells per device until the channel becomes clogged. Deng et al. [82] demonstrated that a throughput of a million cells per minute can be obtained by propelling the cells into a spike-like structure extruding from the channel wall. The biggest advantage of this technique is that it has showcased the scope to transfect a wide range of cargos. However, this technique still has limitations to transfect different cell types, such as stem cells and immune cells. Moreover, compared to other methods, it is a direct membrane poration technique and, therefore, it does not depend on exogenous materials, chemical modification of payloads, or endocytic pathways [43].

**Figure 5:**
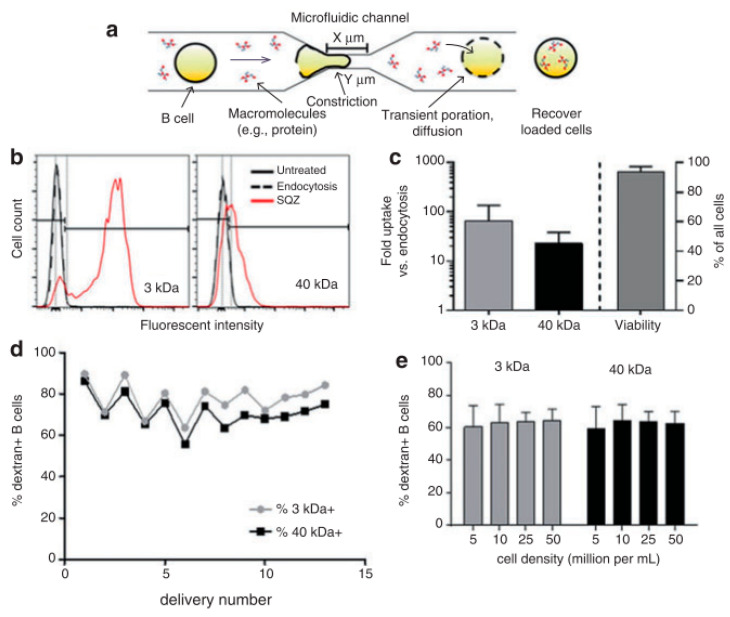
(**a**) Schematic of the cells moving through a micro constriction in the platform. (**b**) Fluorescence intensity comparison between control cells and cells used in the cell squeeze device for transfection of 3-kDa and 40-kDa dextran. (**c**) Cargo uptake study with respect to the number of folds of delivery via endocytosis for 3-kDa and 40-kDa dextran (**d**) Comparison of transfection efficacy for 3-kDa and 40-kDa dextrans. (**e**) Transfection efficacies of 3-kDa and 40-kDa dextrans for various cell densities. Reprinted with permission from ref. [83]. Copyright © 2015 Gregory Lee Szeto et al.

Szeto et al. [83] (2015) proposed a “cell squeezing” technique that disrupts the cell plasma membrane which enables intracellular transfection of whole proteins into B cells from the surrounding medium by mechanoporation (Figure 5). This technique is supposed to be the initial known method of using the microfluidic system to decouple antigen loading from the process of B cell activation. B cells are activated by the binding of antigen to receptors on its cell surface, which causes the cell to divide and proliferate. According to (Castiglioni et al. 2004) [84], mature B lymphocytes (B cells) identify antigens with the help of their B cell receptor and are stimulated to become antibody-generating cells [85]. Cell squeezing provides a system that can prime autologous B cells for in vitro Cytotoxic T lymphocyte (CTL) expansion and helps in facilitating the development of B cell-based vaccines.

By using the cell squeeze [83] technique, a commercial microfluidics and pressure system (SQZ Biotech) was developed for the transfection of whole protein antigen to resting B cells. The chip dimensions are as follows: 30-4 × 1, 10-4 × 1, and 30-5 × 5, (where X-Y × Z denotes Z sequential constriction channels of dimensions X μm long and Y μm diameter). Before the operation, the microfluidics chips and holder were kept in an ice-water bath until they become cold. The cell suspension was sent through the device in 200 μL aliquots at 120 psi. Endocytosis control B cells were prepared identically in medium with Ovalbumin (OVA) but did not go through the microfluidic device. After antigen loading, cells were allowed to rest at room temperature for 5 min and washed twice with PBS. To assess delivery efficiency, fluorescently labeled dextran uptake was measured using flow cytometry. Cytosine photodiester Guanine (CpG) has been shown to improve antigen uptake and activate both mouse and human B cells by Cho et al. [86]. Szeto et al. validated the cytosolic delivery of antigens to the B cells by co-culturing the treated B cells with OVA-specific OT-II CD4+ T cells. No proliferation of the CD4+ T cells was observed. This proved that the material was delivered into the cytosol directly which leads to Major Histocompatibility Complex (MHC) class I presentation and not into endosomal compartments, which leads to the MHC class II presentation pathway. The squeezed B cells were also able to prime antigen-specific CD8+ T cells in vivo. The outcome here can be used to develop similar devices as a platform for cellular vaccines.

#### 3.1.2. Combination of Mechanoporation and Electroporation (Mechano-Electroporation)

**Figure 6:**
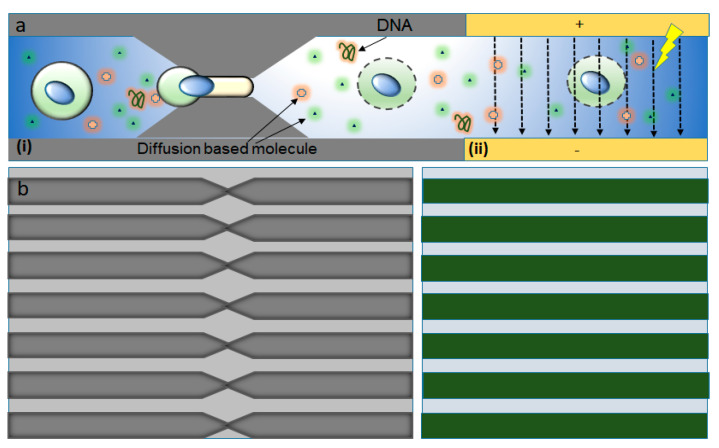
Mechano-electroporation device design and working principle. (**a**) Illustration of the working mechanism (**i**) mechanoporation as the cell moves through the constriction and (**ii**) the electric pulses applied driving DNA into the cytoplasm and nucleus through the disrupted membrane. (**b**) Magnified set of similar parallel microfluidic constrictions etched into a silicon wafer (left) and a set of electrodes deposited on a Pyrex wafer (right). Figure has been redrawn from ref. [87].

Ding et al. [87] developed the design of a microfluidic device where cells are passed at high velocity across the microfluidic constrictions channel, where the constriction channel dimension is lesser than the cell diameter; therefore, the cell membrane is mechanically disrupted. Then a subsequent electric field is applied across the cells to additionally porate the nuclear envelope and thus deliver DNA molecules into the cytoplasm and nucleus (Figure 6). The individual mechanoporation mechanisms, such as cell squeezing, have displayed prominent efficiency, particularly, in the delivery of proteins and nanomaterials with minimal toxicity in multiple cell lines. However, those techniques had not been that successful with DNA delivery, probably due to inefficient nuclear transfection. The authors showed here a drastic improvement in the efficacy of DNA expression and fast nuclear localization through this device. Furthermore, DNA plasmids were transfected successfully to the cytoplasm as well as a nucleus at high throughputs which can go to millions of cells per minute for each device.

A device which comprised of a set of parallel, similar constriction channels and a set of electrodes was developed by Sharei et al. [76]. Similar as the previous cell squeeze design, constriction zones of 75 parallel channels were etched into a silicon wafer with the help of Deep Reactive Ion Etching (DRIE). Then, electrodes were included in the disruption and field enhanced (DFE) device developed by using anodic bonding of Pyrex patterned with electrodes. The width and length of the constriction were in the ranges 4–10 μm and 10–30 μm, respectively. The length, width, and gap space between each electrode were 8 mm, 60 μm, and 40 μm, respectively. This allows a sufficiently high electric field (~2 kV cm^−1^) to be generated with low applied voltage. The duty cycle and period of the electric pulse applied to the device varied from 50 to 500 s and from 1% to 10%, respectively, consistent with values usually used for electroporation. The DFE device was operated at a throughput of 100,000–500,000 cells s^−1^ per chip per run and every run took 5–20 s. Various parameters governed the performance of this device and they are (1) cell speed, (2) constriction dimension, (3) electric pulse profile, and (4) strength and the number of pulses. Several experiments were performed to characterize DNA transfection with the DFE technique by treating a mixture of HeLa cells and green fluorescent protein (GFP) plasmid DNA at different pulse amplitudes. These experiments used a device with a constriction length of 10 μm and a constriction width of 6 μm, which was known as a 10–6 DFE device. The results showed that cell transfection reached above 60% when the applied amplitude increased to 8 V and 90% when the amplitude was 10 V. The influence of cell speed on the transfection was also investigated. When a pulse of 10 V was applied, the DNA expression decreased when cell speed increased because of the reduced number of pulses the cell received as it passes through the electric field. The cell viability and DNA expression seem to have the best balance at cell speeds of around 300 mm/s. It was also observed that severe electrical damage would occur at low speeds and mechanical damage at high speeds. The results show that DNA and mRNA are significantly dependent on the application of the electric field, while protein delivery is more dependent on mechanical membrane disruption methods. Thus, Ding et al. [87] demonstrated that mechanical forces and electric field effects can be combined to accomplish reversible nuclear and plasma membrane poration for both nuclear and cytosolic transfection.

### 3.2. Electroporation

Direct penetration methods deliver the external molecules into the cytoplasm directly, but field-induced membrane poration methods create pores by disrupting the plasma membrane transiently so that molecules can enter the cells through the pores by the simple diffusion process. Electroporation is one of the most widely adopted physical methods for intracellular delivery both in vitro and in vivo [43,88,89].

Theoretically, electroporation is the formation of pores in a membrane due to the application of a potential difference across that membrane [45,90]. When the potential difference reaches a critical value, the chances of electroporation taking place drastically increases. The major parameter of electroporation is the trans-membrane potential although there is no fixed voltage threshold that initiates electroporation. Experiments were conducted for a thorough study of the interconnection between the transmembrane potential and pore formation. The theoretical forecast displayed that disruption happens only to the membrane area where trans-membrane potential is above a critical threshold range of around 0.2 V–1.0 V [91,92].

**Figure 7:**
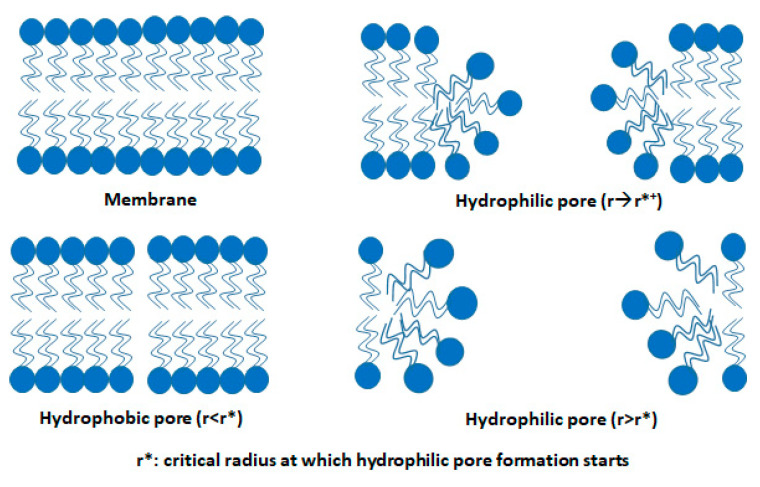
Rearrangement in the structure of the lipid bilayer membrane during electroporation. Figure has been redrawn from ref. [45].

Generally, cell membrane structure is a phospholipid bilayer membrane and typically possesses a thickness of ~5 nm [93]. One layer of phospholipid consists of a polar head and non-polar hydrophobic hydrocarbon tails. The head and tail are designed in such a manner that the polar head of the two layers face opposite to one another and hydrophobic tails remain at the middle part of the membrane structure which makes them amphiphilic (Figure 7). Due to the application of an external voltage pulse, the cell membrane behaves akin to a dielectric capacitor [45]. The field is highly intense (normally in the order of 10^3^–10^8^ V m^−1^) across the membrane depending on the experimental environment [92,94]. Thus, a strong electrical force acts on the membrane which leads to pore formation [95,96,97].

In the traditional electroporation method, an electric field is applied to a group of millions of cells located between the electrodes, called Bulk Electroporation (BEP) [90]. In this method, precise biophysical studies in a single cell are not possible as there is no control over individual cells. Further, BEP has its limitations as it creates side effects such as acute joule heating, substantial pH change of solutions, and lack of precise delivery [98,99], etc. The single-cell electroporation (SCEP) method showcases large transfection of individual cells ranging from a single cell to high throughput with a highly focused electric field induced by a few volts [56,88,100].

Moreover, SCEP methods manipulate cell positioning, cell concentration, and reduce reagent consumption. The SCEP offers qualitative and quantitative control to conduct cell-autonomous protein function studies which are not possible in BEP [101]. Furthermore, the materials of single cells can be extracted and studied. This analysis is important for studying the distribution of different critical functions between cells, unlike mean data analysis from a huge group of cells in BEP [102,103]. The cells which are similar in various parameters such as dimensions, content, and general characteristics show the difference in cellular response and gene expression. The SCEP mechanisms have become more popular in the research community because of their robust and simple working mechanism to study any kind of cells for discovering bio-physical properties as well as different omics [104]. This implies that BEP can provide average data, but is not the appropriate method to explain accurate cellular function at the single-cell level [105]. We will discuss some of the single-cell electroporation devices fabrication, mechanism for intracellular delivery, and analysis.

#### 3.2.1. Microfluidic Electroporation

**Figure 8:**
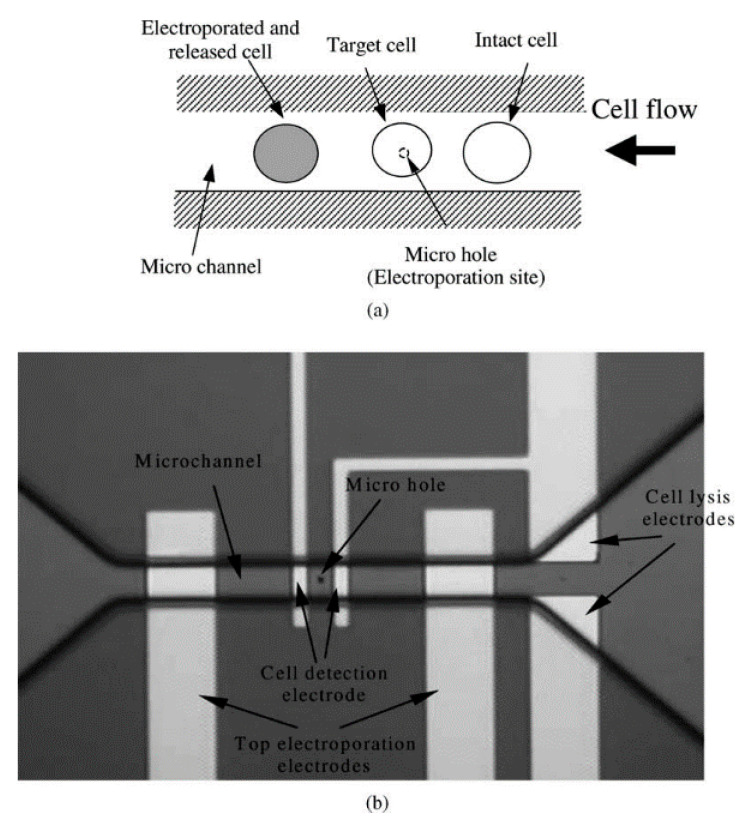
(**a**) Principle of flow through the micro-electroporation chip (**b**) Layout image of micro-hole, micro-channel, and integrated electrodes. Reprinted with permission from ref. [107]. Copyright © 2003 Elsevier.

One of the initial SCEP systems was developed by Huang and Rubinsky [106] in the late 1990s. It was a small hole of 2−10 μm diameter that a single cell could be drawn into. An electric pulse applied from below to disrupt the trapped cell and the mechanisms of electroporation at the single-cell level were studied. Although it was only demonstrated as a proof of a concept, such developments motivated the field toward more improved efforts. After several years, the first microfluidic flow electroporation devices were developed by Huang and Rubinsky [107]. They demonstrated the loading of small molecule dyes and transfection with GFP-encoding plasmids at low throughput. The device was able to transport the cells to the electroporation region by a microfluidic channel in a precise manner, which led to a 100% manipulation rate (Figure 8). The ND-1 cell and EGFP gene were successfully delivered to verify the feasibility of single-cell manipulation.

#### 3.2.2. Nanochannel Electroporation

**Figure 9:**
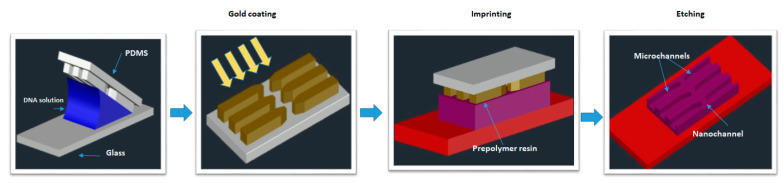
Design and operation of a NEP platform. The Fabrication process of the NEP device. Schematic of the PDMS lid on the NEP chip. The nanochannel has a diameter of 90 nm and length of 3 µm. Figure has been redrawn from ref. [108].

The membrane disruption effect using electroporation could be concentrated on a very small area of the cell surface by scaling down the size of the aperture. This technique of poration is known as nano-electroporation (NEP). A prominent advantage of this strategy is the control of the dose, i.e., the finding that the amount of delivered material directly correlates with the duration of the voltage pulse. Nanochannel electroporation also seems to introduce agents faster and deeper into the cytoplasm as a result of enhanced and concentrated electrophoretic forces. Compared to conventional electroporation and other forms of microfluidic electroporation, it was proposed that the nanochannel delivery technique was based on electrophoretic forces rather than diffusion and/or endocytosis. Nanochannel electroporation was successful in delivering dyes, oligonucleotides, small interfering Ribonucleic Acid (siRNA), plasmids, and quantum dots into the target cells. One major thing to be noted is that before electroporation, every single cell required placement against the nanochannel with optical tweezers. Boukany, P., Morss, A., Liao, Wc. et al. [108] designed a NEP device with a nanochannel between two microchannels to transfect the biomolecules into the cell precisely, without affecting the cells. An optical tweezer is used to place the cell on one microchannel of the platform (Figure 9). When an electric pulse is applied between the two microchannels, the delivery of molecules into the cell occurs. The authors validate that none of their experiments resulted in cell death, as the damaged area of the cell membrane is very limited in NEP.

#### 3.2.3. Nanostraw Electroporation

**Figure 10:**
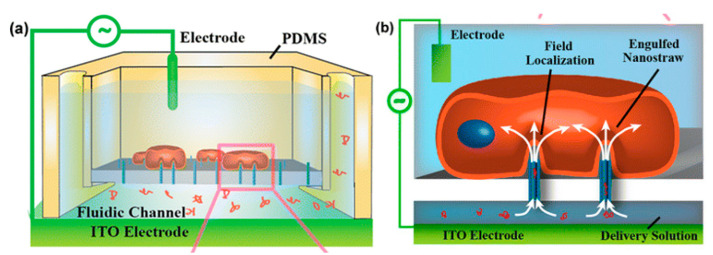
(**a**) Schematic diagram of nano-straw-electroporation platform. (**b**) Schematic of field localization and confinement of biomolecule at the nano-straw tip. Reprinted with permission from ref. [109]. Copyright © 2013 American Chemical Society.

Another method of nanoscale single-cell electroporation takes place in the form of nano-straws. The major difference is that the nanoscale aperture injects into the target cell akin to a hollow nanoneedle. Although under passive conditions cell membranes seem to be opposed to penetration by such nanoneedles, the application of an electric field creates pores on the cell membrane at the tip of the nano-straw. Xie et al. [109] introduced a robust nano-electroporation device made on alumina nano-straws coming out of a track-etched membrane and generates an array of hollow nanowires connected to a bottom microfluidic channel (Figure 10). One advantage of this approach is that optical tweezers or positive dielectrophoresis are mostly not required to establish optimal contact between cells and the nano-straw. In addition, if cells properly adhere to the nanostraw array, substantial pumping forces can presumably be used to flow molecules into the cytoplasm without cell detachment.

#### 3.2.4. Nanofountain Probe Electroporation

**Figure 11:**
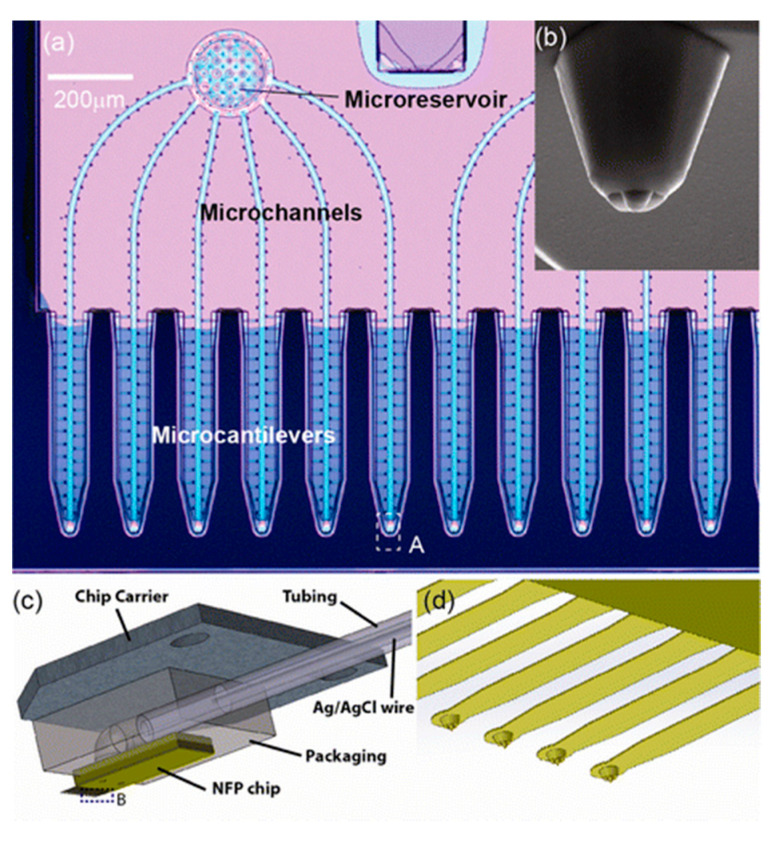
Nanofountain probe device: (**a**) optical microscopy image of the NFP with multiple probes for parallelized single-cell electroporation; (**b**) SEM image of a view of the probe; (**c**) Illustration of the packaged NFP chip; (**d**) magnified view of the cantilevers in region B of (**c**). Reprinted with permission from ref. [101]. Copyright © 2013 American Chemical Society.

A scanning probe-based approach for single-cell electroporation, called nano-fountain probe electroporation, has been introduced by Espinosa et al. [101,110]. It is primarily an atomic force microscope cantilever engineered with a hollow channel for fluid flow. A grounded coverslip is used to culture the target cells and voltage is applied to the conductive cantilever. This helps in focusing the electric field at the site of contact between the cell and cantilever (Figure 11).

By monitoring the motion of the fluid tip and the flow, dextrans and proteins can be introduced into cells. As the follow-up of the system, it has been utilized to transfect molecular beacons to the cytoplasm for the diagnosis of mRNA transcription successfully [110].

#### 3.2.5. Micro/Nano-Electrode-Based Devices

**Figure 12:**
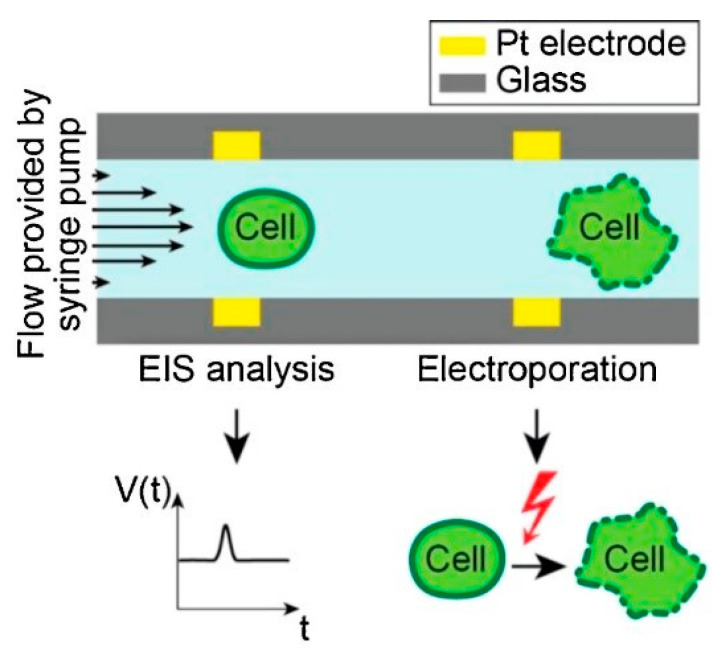
Design and working principle of the microchip. Reprinted with permission from ref. [111]. Copyright © 2015 Elsevier.

Bürgel et al. [111] in 2015, proposed a microchip where Pt electrodes of thickness 200 nm facing each other were modeled across a microfluidic channel, as shown in Figure 12. The device is combined with electrical impedance spectroscopy (EIS) that provides a label-free study to understand the type, size, and dielectric properties of cells. In EIS, an AC voltage is applied using a pair of electrodes. A current flow is generated between them which affects the passing of cells between the electrodes. The change in current is observed to determine the dielectric properties of the cell. During the passage of a single cell, the electrodes were temporarily switched on.

Zhang et al. [112] developed co-planer microelectrodes, which are made in the sidewall of micro-cavity geometry on the microchannel of the microfluidic chip. It can generate electroporation around the contact area of cells and cavity resulting in high yield electrofusion in Myoblast cells. To probe pore distribution for various configurations of electrode microcavity structures, a theoretical study was conducted along with experiments.

**Figure 13:**
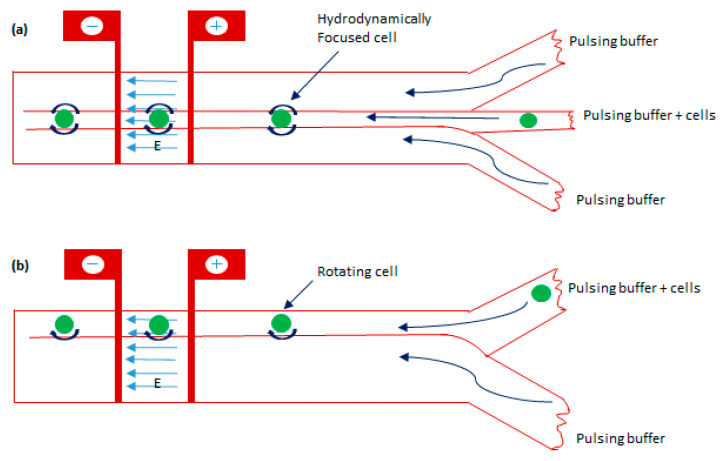
The schematic diagram of the electroporation technique is based on cell rotation with two configurations. (**a**) A microchannel having three inlets with cell carrier flows being hydrodynamically focused between two sheathing flows. (**b**) A microchannel having two inlets with electroporating electrodes across the channel. Figure has been redrawn from ref. [112].

In the above-discussed micro/nanodevices, the cell membrane is electroporated only across the fraction of the cell surface which is perpendicular to the electric field distribution. Due to this, the only fraction of the cell surface gets electroporated and restricts the total electroporation area of a cell. To solve this limitation, Zheng et al. [112] developed a microfluidic device based on the pinch flow technique which can control cell rotation that allows the exposure of the perpendicular electric field all over the cell surface. The device includes a two/three-inlet microfluidic channel arranged with a pair of micro-electrodes with an inter-electrode distance of ~410 microns. The schematic diagram of the device is shown in Figure 13.

**Figure 14:**
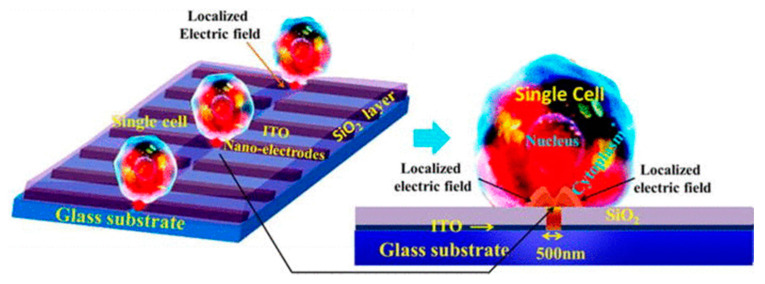
Schematic diagram of LSCMEP platform. The applied electric field porates a small area of the cell membrane for delivery of the drug into the cell. Reproduced with permission from ref. [113]. Copyright © 2014 The Royal Society of Chemistry.

Santra et al. [94] proposed a localized single cell nano-electroporation with various transmembrane potential (TMP) values in both reversible and irreversible poration techniques. Lower TMP values generate low-density pores on the membrane which results in slow transfection of biomolecules into the cell. However, extremely high TMP values generate a larger density of pores on the cell membrane and results in the fast delivery of molecules, which may affect the membrane badly. To resist this, the transfection rate is optimized by controlling the TMP values. Santra et al. [113] has also performed controlled molecular delivery using the single-cell nano-electroporation method by millisecond to nanosecond electric pulses. In this case, a pair of transparent indium tin oxide (ITO) based nano-electrodes were used to generate an electric field (Figure 14). The ITO microelectrodes with submicron gaps were fabricated by Chen et al. [114] using a focused ion beam (FIB) technique.

#### 3.2.6. Field-Effect Transistor-Based Device for SCEP

**Figure 15:**
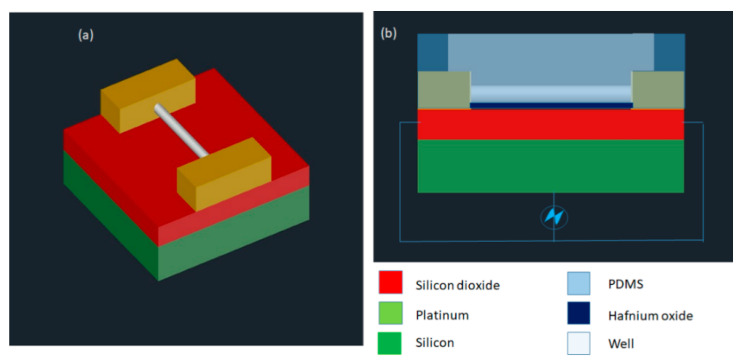
(**a**) Device design is shown in the diagram. (**b**) Electrical schematic diagram of the device. Figure has been redrawn from ref. [115].

Nanowire and nanoribbon channel-based field-effect transistor was used by Jokilaakso et al. [115] for SCEP. Silicon nanowires with diameters in the range of 50–100 nm or nanoribbon with width ~2 µm as transistor channels were used to generate a highly specific electric field above the surface of the device. The cells were moved by magnetic beads into the desired position and thus negates the need for any cell trapping system. In this study, pulsed voltages were provided between the shorted source drain and the back gate of the transistor. It was also shown that the cell at the top of the transistor was only lysed (Figure 15). The most adjacent cells remained unaffected and thus allowed for single-cell transfection. A hundred percent of the cells were seen to be lysed at 900 mVpp voltage for 1 s.

#### 3.2.7. Nano-Localized Electroporation

**Figure 16:**
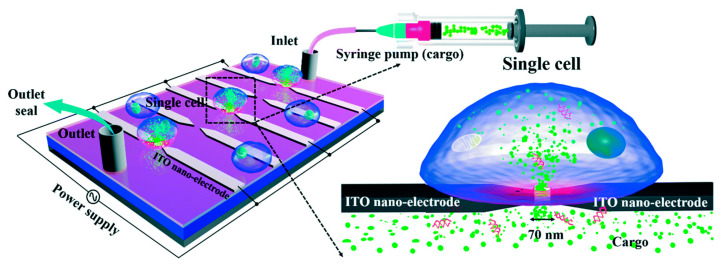
Schematic diagram of the nano-localized single-cell nano-electroporation platform. Reprinted with permission from ref. [116]. Copyright © 2020 The Royal Society of Chemistry.

Santra et al. [116] developed a nano-localized single-cell nano-electroporation device that comprised of ITO nano-electrodes in a triangular-shaped array (Figure 16). The nano-electrodes have a tip diameter of 40 nm and a gap of 70 nm between two electrodes. A strong electric field is generated by applying a voltage between the nano-electrodes. This disrupts various nano-localized points of the cell membrane which helps in delivering biomolecules without loss in cell viability. This new method provides high cell viability (~98%) and high transfection efficacy (<96%) concerning delivering various biomolecules into single cells. Santra et al. [117] designed another nano-localized single-cell nano-electroporation device that has a triangular tip of 40 nm and a gap of 60 nm between two nano-electrodes. The electric field generated was used to disrupt the membrane of a single HeLa cell for biomolecular delivery.

The SCEP techniques demonstrated above have their limitations also. For example, the microelectrode EP method provides single-cell selectivity by handling the microelectrodes individually. However, this is not a direct method of cell selection. The micropipette-based mechanism depends on the researcher’s selection of a specific cell from a population. Moreover, the technique of disrupting cells one by one is very slow and needs a skilled technician, in addition to having a low throughput [118,119].

#### 3.2.8. Parallel Single-Cell Electroporation

As an alternative or modification of the above-discussed approaches, in situ parallel SCEP approach was first demonstrated by Valley et al. [120] in 2009 in which intracellular delivery was performed in an array of single cells at the same time with control over each cell. The device was combined with optical tweezers to monitor parallel single-cell transport.

**Figure 17:**
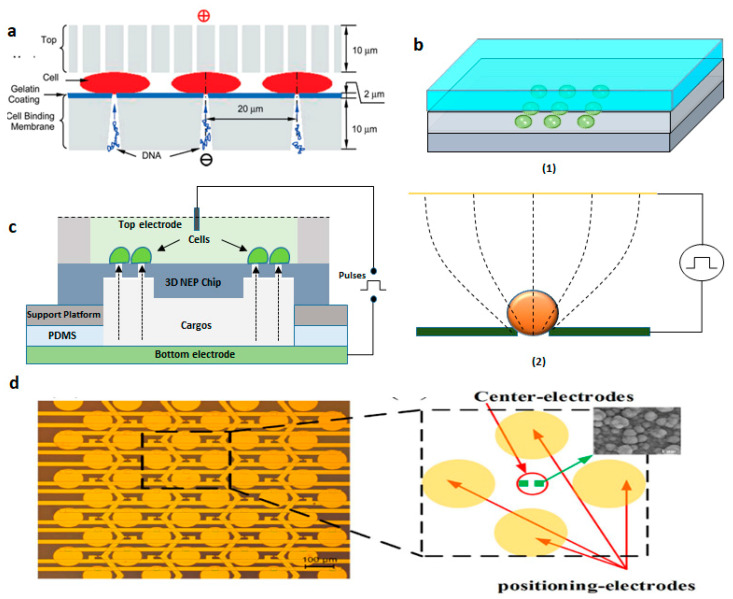
(**a**) Schematic of MSE disk setup. Reprinted with permission from ref. [121]. Copyright © 2010 American Chemical Society (**b**) (**1**) DEP device configuration and (**2**) The principle of electroporation. Figure has been redrawn from ref. [122]. Copyright © 2010 IEEE (**c**) Schematic of 3D Nanoelectroporation device. Figure has been redrawn from ref. [123]. Copyright © 2010 The Royal Society of Chemistry (**d**) Device design of the micro-electrode array chip. Reprinted with permission from ref. [125]. Copyright © 2016 Xiaoliang Guo et al.

Fei et al. [121] exhibited micro nozzle array structures to enhance non-uniform electric field distribution on a cell population which is, indeed, parallel SCEP. This allowed the manipulation of cell distributions and controlled electroporation of a bunch of cells. Experiments were conducted on mouse embryonic stem cells to deliver with plasmids gWiz SEAP as reporter genes, and transfection of the 100 × 100 arrays was evaluated 24 h after electroporation. The micronozzle structures were delivering molecules around twice compared to microchannels because the electric field is more focused at the small end of the micronozzle. This resulted in better-localized electroporation resulting in ~75% cell survival (Figure 17a). Wang et al. [122] independently developed a SCEP chip that can locally electroporate a cell array with an operating voltage as low as ~1 V. The chip contains one bottom metal electrode on the top of a substrate, a sandwiched dielectric polymer layer with thickness ~10 µm patterned with an array of microwells, and a conducting top lead as another electrode. The gap between these two electrodes was 60 µm, the diameter of the microwell was ~10 µm (Figure 17b). Human lymphoma cells in an 8 × 11 array were electroporated successfully with ~100% electroporation yield. This method could negate bubble occurrence because of the electrolysis of water and joule heating due to the high field strength achieving both DEP trapping and cell electroporation with the same device.

The three-dimensional Nanochannel electroporation chip (NEP) was proposed by Chang et al. [123] in 2016 which is capable of electroporating ~40,000 cells per cm^2^ of the chip (Figure 17c). This system is comprised of four components: a 3D electroporation chip, a support platform, a PDMS spacer, and a pair of electrodes. There is a PDMS chamber at the top of the 3D chip to load cells to be electroporated. The cargo molecules are transported into the bottom chamber, which is separated from the top chamber by the 3D chip. The device exhibited approximately > 90% of cell viability and ~90% of transfection efficiency. Precise control overdosage with high throughput is offered by this method. In the follow-up study, they updated the NEP chip with a micro-cap array to align the cells on nanochannel in a much more precise manner [124]. A massively high-throughput parallel delivery was obtained of genetic cargo (microRNA, plasmids) into mouse primary cardiomyocytes with delivery efficiencies at ~90–100% in a controllable manner.

Guo et al. [125] designed a microelectrode array chip that has the features of cell positioning and real-time impedance monitoring for cell-selective parallel electroporation at a single-cell level. It consists of a quadrupole-electrodes unit (positioning electrodes) and a pair of planer electrodes (center electrodes) located at the centers of each quadrupole-electrode unit for conducting electroporation (Figure 17d). Using the positions, the cells were trapped and positioned onto the center electrodes based on negative dielectrophoresis. Lastly, the electroporation was performed by applying a voltage to the center electrodes. An efficacy of greater than 90% was obtained by selective electroporation.

The commercial use of the above-discussed methods requires robust usage techniques with good reliability and performance. In this century, robust systems with high-level automation are encouraged for electroporation approaches. Moreover, the entire operating system has to be combined into one single system to avail of the complete scope of SCEP [45].

### 3.3. Optoporation

In recent years, photo or optoporation is one of the most emerging physical methods for intracellular delivery. In this approach, light energy is concentrated onto a cell membrane in the presence or absence of nanoparticles to generate micro or nanobubbles, which breaks the cell membrane. This creates transient membrane pores to transfect molecules into cells [126,127,128,129,130]. Membrane disruption can be enhanced either with the help of a tightly focused light beam (pulsed or continuous wave laser) or by a broad light beam integrated with sensitizing nanoparticles [131].

An optoporation technique was reported by Tsukakoshi et al. in 1984 to transfect membrane-impermeable molecules into a cell by generating pores with the help of a pulsed laser [132]. Nikolskaya et al. adopted a continuous laser to transfect fluorescent molecules in neonatal rat cardiac cells. This research exhibited a high target specific delivery and high spatial resolution by optoporation [133]. Andrew et al. utilized a femtosecond pulsed laser for the disruption of Chinese hamster ovarian cells [134]. The pulsed lasers gave better energy efficacy and lesser cell death over continuous lasers for optoporation.

Femtosecond (fs) lasers can generate sub-micrometer-sized holes in the targeted single-cell membrane by multi-photon approaches and production of a low-density plasma at the cell surface [135,136,137,138,139]. The delivery efficiency utilizing femtosecond-laser poration can go up to 80% to 90% [138,139]. Nanosecond (ns) lasers can also disrupt cells around laser-induced cavitation bubbles but causes heating and thermos elastic stresses on the adjacent cells [139]. Furthermore, the effective zone of nanosecond laser poration is too large for targeting single cells [140]. Moreover, the cell membrane is heated by continuous-wave lasers to form permeable pores with less than 30% transfection efficiency [46,141]. To further improve the coupling efficacy of photonic energy into cell poration, nanoparticle-mediated methods have been proposed [128,142,143].

**Figure 18:**
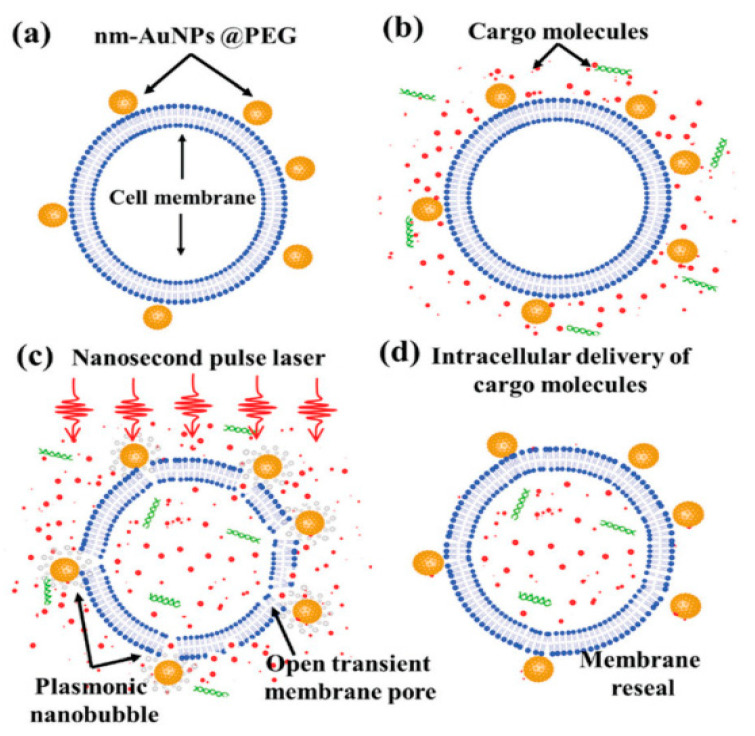
Schematic overview of photoporation procedure for intracellular delivery: (**a**) cells was incubated with PEG mediated nm-AuPNPs for attachment onto the cell membrane; (**b**) cargo molecules were added just before laser exposure; (**c**) nanosecond pulse laser-induced plasmonic nanobubbles formation at nm-AuPNPs and cell membrane interface resulting in transient pore formation; (**d**) successful intracellular delivery of cargo molecules with membrane reseal. Reprinted with permission from ref. [128]. Copyright © 2020 The Royal Society of Chemistry.

**Figure 19:**
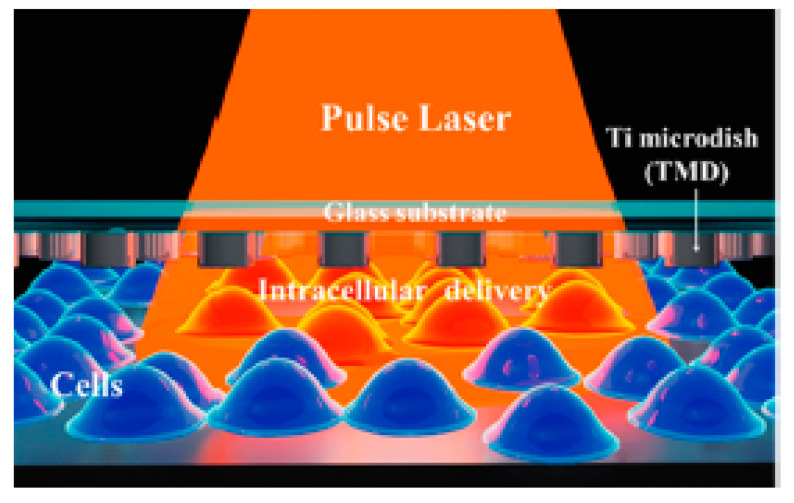
Schematic of the parallel high-throughput intracellular delivery platform using the photoporation technique. A TMD microdevice was exposed to a pulse laser with an Infrared (IR) wavelength and photothermal cavitation bubbles were generated to disrupt the plasma membrane and deliver biomolecules into the cells. Reprinted with permission from ref. [129]. Copyright © 2020 American Chemical Society.

Santra et al. [128] reported effective intracellular transfection of molecules with high cell viability with the help of nanosecond-pulsed laser-activated plasmonic photoporation. This is intervened by high-aspect-ratio nano corrugated mushroom-shaped gold-coated polystyrene nanoparticles (nm-AuPNPs) at near-infrared wavelength. On application of the laser, nm-AuPNPs demonstrate greater plasmonic extinction than spherical AuPNPs because it enhanced their energy efficacy and shortened the required illumination of light, efficiently controlling cell damage and enhancing transfection efficacy. Nm AuPNPs demonstrate surface plasmon absorption at the near-infrared region with a peak at 945 nm. Pulsed laser application at this plasmon peak stimulates explosive nanobubbles, which disrupts the cell membrane, allowing the delivery of dyes, quantum dots, and plasmids into the cells (Figure 18). However, this method demonstrated the bulk delivery approach with high delivery efficiency and cell viability without using a microfluidics platform. Another method [129] of nanoscale photoporation takes place in the form of an array of titanium micro dish (TMD) (Figure 19). The device avails Near Infrared (NIR) radiation to rupture the plasma membrane by generating photothermal cavitation bubbles thereby delivering biomolecules into the cell. SiHa cells provided better results with 96% delivery efficiency and 98% cell viability for propidium iodide; 98% delivery efficiency and almost 100% cell viability for dextran 3000 MW; and 92% delivery efficiency and 98% cell viability for dextran 10,000 MW.

**Figure 20:**
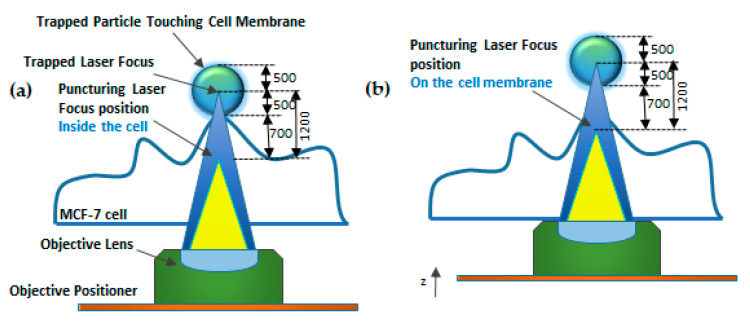
Schematic diagram of Focused Laser Beam Single-cell Optoporation. (**a**) 1064 nm laser (blue) as an optical tweezer to trap particle. (**b**) An 800-nm femtosecond laser (yellow) used as a puncturing laser for membrane disruption. Figure has been redrawn from ref. [144].

Waleed et al. [144] demonstrated single-cell intracellular delivery of MCF-7 cells with pAcGFP1-C1 plasmid coated 1 µm amino-based polystyrene microparticles with the help of a femtosecond laser (800 nm) integrated with optical tweezers (1064 nm) (Figure 20). The cellular membrane of the MCF-7 cancer cells was disrupted at a single point with the help of a precisely focused near-infrared (NIR) femtosecond laser pulse. Optical tweezers are used to trap the microparticles to tightly focus the femtosecond laser on the plasma membrane to disrupt it. This method provides control over the amount of plasmid that is delivered into a cell, high selectivity, and aseptic conditions because of the non-contact nature of the approach. The major disadvantages of this method are the low transfection efficiency of 12.7% and the low transfection frequency of 20 cells per hour, which makes it not feasible for practical applications.

**Figure 21:**
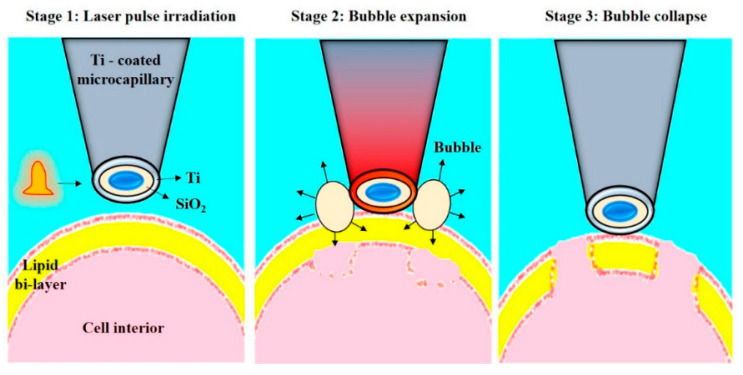
Schematic representation of the Nano blade. The figure depicts the process of cell membrane pore formation by creating microbubbles. Reprinted with permission from ref. [146]. Copyright © 2010 Optical Society of America.

Wu et al. [145] designed another single-cell intracellular delivery mechanism using metal light interaction-based nanobubble, which generated shear stress on the cell membrane (Figure 21). They designed a nano blade by depositing 100 nm titanium on a glass micropipette. This nano blade is held near the cell surface. A pulsed laser is applied to the titanium nanostructure. As a result of the Laser-Induced Plasmon Effect (LSPR), microbubbles are generated, which break the cell membrane to produce transient hydrophilic membrane pores [146]. With the help of this approach, they pushed the cargo into the cell through a micropipette with 46% transfection efficiency and 90% cell viability.

**Figure 22:**
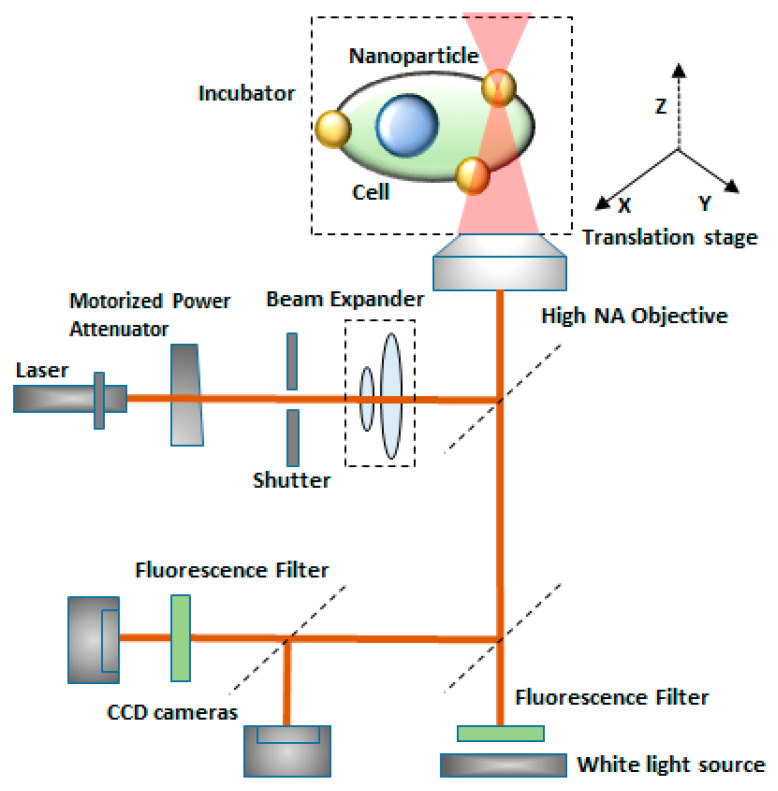
Schematic diagram of the experimental setup for single-cell optoporation. Gold nanoparticles are markers of the live cell membrane position which enhances local pulsed-laser optoporation. Figure has been redrawn from ref. [147]. Copyright © 2020 Royal Society of Chemistry.

Sergiy Patskovsky et al. [147] proposed an optical platform for investigating the mechanism of nanoparticle-assisted pulsed laser-based single-cell optoporation. Plasmonic nanoparticles (NPs) were considered as markers of the exact spatial position of living cell membranes. Moreover, they enhance the localized pulsed laser-activated cell disruption. The NPS images of high contrast were obtained by reflected light microscopy (RLM), which provides accurate and automatic laser targeting at each NP for spatially controlled laser-based single-cell optoporation at a specific point. This method helps in the study of real-time perforation kinetics of live cells and the optomechanical interaction of NPs with membranes, due to its compatibility with fluorescence microscopy and a cellular incubator (Figure 22).

The Optoporation technique has garnered significant levels of attention over the last several decades, particularly for cellular transfection and analysis. However, one of the main problems is the high cost of lasers and the optical systems required to operate them, as well as the poor adoption outside of expert communities. A second main problem is the question of how to increase the throughput of treatments, which is an area, where microfluidics and computer automation have made positive contributions. Future progress in optoporation is likely to require creative ideas to move beyond traditional limitations.

### 3.4. Sonoporation

Sonoporation is a physical approach of membrane disruption, in which an ultrasound is applied in the presence of microbubbles that acts as cavitation nuclei, which forces the cell membrane resulting in disruption and the creation of transient membrane pores to allow biomolecules into the cell [148]. The sonoporation includes ultrasound contrast agents, to allow the delivery of cargo molecules into cells. It is a transient and reversible phenomenon that obtains high efficiency as well as high cell viability. A critical limitation of sonoporation is gene delivery, where ultrasound parameters need to be optimized to obtain high gene transfection efficiency and to maintain good cell viability [149].

Sonoporation is primarily performed for the bulk treatment of large cell numbers in vitro or tissue volume in vivo. It is facilitated by microbubbles that are either injected in the vasculature or solution with suspended or attached cells. The application of ultrasound generates cavitation microbubbles, which are marked by rapid volume contraction/expansion and/or collapse.

Cavitation bubbles expand and shrink in response to the low-and high-pressure portion of the ultrasound wave. If the resultant oscillation in the bubble size is stable (repeatable over many cycles), it is called “stable” or “non-inertial cavitation”. A circulating fluid flow (microstreaming) [150,151] is generated by such oscillations around the bubble with velocities and shear rates proportional to the oscillation amplitude. The associated shearing forces created at high amplitudes can deform and disrupt the synthetic vesicles [150]. Thus, the ultrasound-mediated transfection of drugs and genes is a complex process with the interplay among target tissue, therapeutic agent, nature of ultrasound energy, and characteristics of microbubbles.

However, at the single-cell level, the processes related to Sonoporation mediated transmembrane and transcellular transport are not understood precisely. The absence of such clear understanding becomes a hindrance to the successful technological development of this technique as an effective strategy. The lack of precise control and real-time assessment of the transient and microscopic process of single-cell Sonoporation is considered to be the most prominent challenge for quantitative investigation. The complex nature of the microbubble cavitation shows great difficulties to control Sonoporation at the single-cell level.

**Figure 23:**
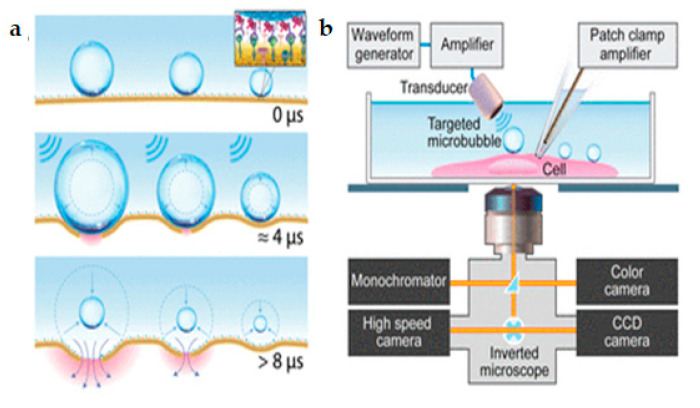
(**a**) Ultrasound excitation of microbubbles attached to the cell membrane via receptor-ligand binding. (**b**) Experimental setup for real-time assessment of single-cell Sonoporation using synchronized simultaneous patch-clamp recording, bright field video microscopy, and fluorescence imaging. Reprinted with permission from ref. [152]. Copyright © 2012 Proceedings of the National Academy of Sciences.

Z. Fan et.al. [152] proposed an innovative method for the control and quantification of cell membrane disruption by Sonoporation at the single-cell level. Ultrasound excitation of microbubbles helps in spatially and temporally controlled membrane disruption with high repeatability (Figure 23a). The microbubbles were targeted towards the HEK-293 cell membrane. Time-resolved determination of single-cell Sonoporation and quantification of the size of pores were obtained using whole-cell patch-clamp recording integrated with fluorescence microscopy (Figure 23b). Controlled transfection with subcellular accuracy and calcium signaling in targeted cells was gained by selective excitation of microbubbles. Finally, Sonoporation was used to transfect calcein into HEK-MRP1 cells and the calcein transport was monitored by MRP1.

Several studies have been conducted to study the challenges mentioned above by developing innovative techniques for creating and trapping a single microbubble for single-cell Sonoporation [49]. However, this method needed complex laser technology for controlling bubbles and interacted with only a single cell at a time. Moreover, these studies were not able to quantify membrane poration and delivery.

### 3.5. Magnetoporation

**Figure 24:**
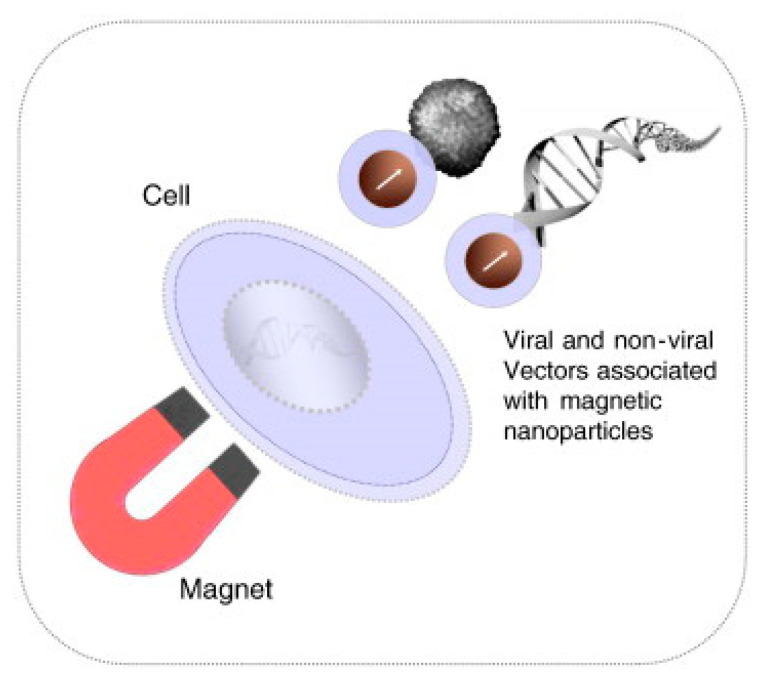
Schematic diagram of the Magnetofection process. Reprinted with permission from ref. [159]. Copyright © 2011 Elsevier.

The magnetoporation is a method of transfecting molecules into the cell under the influence of the magnetic field [153]. A biomolecule-magnetic reagent complex is formed by mixing the cargo molecules with the magnetic nanoparticle. Then the complex is transfected into the cell through the pore created with the help of a magnetic field force [47,154,155]. However, the method of magnetoporation is considered similar to electroporation. It is believed that an electric field is induced by the magnetic field which affects the transmembrane potential of the cell membrane. The cell membrane is disrupted when the transmembrane potential crosses the threshold value (Figure 24). Magnetofection has been capable of delivering difficult-to-transfect molecules into the cells. Neuron cells are difficult to deliver under the influence of a static magnetic field [156]). In this case, the oscillating magnetic fields were designed by Adams et al. [157] to deliver the neural stem cell and because of this two-fold delivery efficiency was achieved.

Various research on magnetoreception techniques has been conducted by scientists to implement the transfection suited for single-cell analysis so that the DNA or other vectors can be delivered effectively to every diseased cell. Igor Medina et al. [158] proposed a magnetoreception-based protocol for delivery of complementary Deoxyribonucleic acid (cDNA) and RNA constructs into rat hippocampal neurons which was cultured in vitro for some hours to 21 days. Various factors that can affect transfection efficiency such as expression levels after transfection time, age of cells during delivery, and factors for double delivery were determined. Furthermore, this process made double-transfection of DNA into small numbers of hippocampal neurons and long-term expression of DNA and short hairpin deoxyribonucleic acid (shRNA) constructs were achieved without influencing neuronal differentiation. This process utilizes a combination of mixed hippocampal cultures, CombiMag, transfection agent, and a magnetic plate; displays low toxicity, and a reasonable efficacy in neurons (ca. 5%) to promote the study of single-cell.

**Figure 25:**
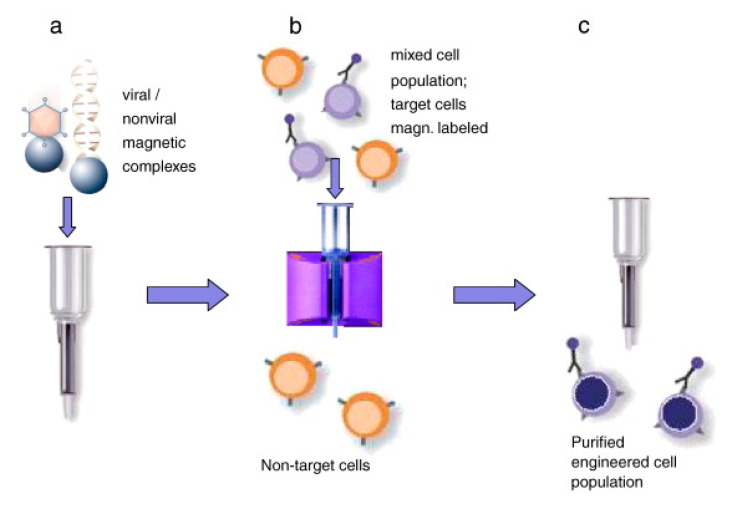
Magselectofection. (**a**) A cell sorting column activated magnetically is loaded with magnetic vectors. (**b**) A high gradient magnetic field is used to associate magnetically activated cells with magnetic vectors. (**c**) The cell sorting device is withdrawn from the magnetic field and selected cells are pushed out from the device. Reprinted with permission from ref. [159]. Copyright © 2011 Elsevier.

Sanchez-Antequera et al. [160] developed a method named Magselectofection that would help in implementing magnetoporation in an automated cell separation device (Figure 25). In this process, (1) the magnetic transfection/transduction process was used to load a commonly used magnetic-activated cell sorting column and (2) under the effect of a high-gradient magnetic field the magnetically-labeled cells were linked with immobilized magnetic vectors. This would result in high transfection efficiency due to improved internalization and contact of the magnetic vectors in the cells [159,160]. This method is effective specifically because it helps in genetic modification and cost-effective separation in a single cell system with a lesser number of handling steps and low vector consumption.

Based on the magnetic properties of multi-walled carbon nanotubes (MWCNTs), an innovative application was proposed by Liu et al. for enhanced cancer cell disruption [161]. Cell membrane disruption was improved using weak magnetic fields of MWCNTs coated by a polymer. These MWCNTs form rotating bundles on the application of magnetic fields [162]. The outcomes of this study have huge potential in clinical applications which include enhanced tumor cell disruption for targeted cancer chemotherapy and mechanical disruption of tumors.

### 3.6. Thermoporation

In recent years, the thermal membrane disruption by heating of single cells has gained attention for cell poration [163], gene expression [164], nano surgery [165,166], migration of cells [167], cell fusion [168], and neuronal spikes generation [169,170]. Few of these researchers were stimulated by a temperature rise at the cellular level utilizing a laser under a microscope. However, the primary limitation occurring at this cell poration technique is the decreased reliability in the measurement of temperature rise in a complex medium such as cytoplasm.

**Figure 26:**
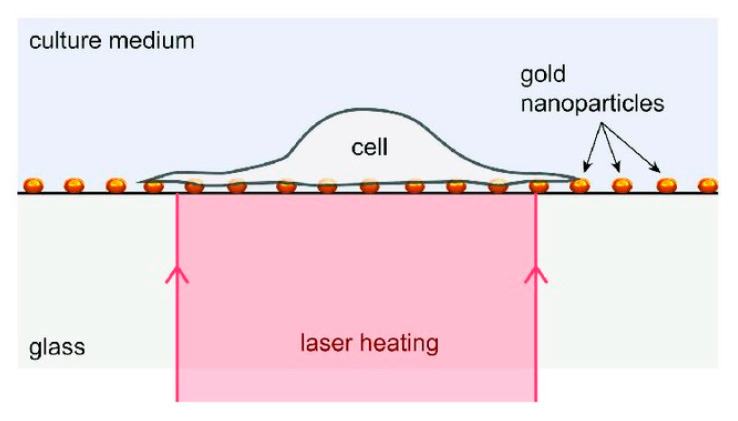
Schematic diagram of the device: RPE cells attached to a layer of gold nanoparticles acts as heat sources under laser activation. Reprinted with permission from ref. [171]. Copyright © 2018 John Wiley and Sons.

Robert et al. [171] proposed a laser-based dynamically controlled heat shock response at the single-cell level, where they used gold nanoparticles, which absorb light, to act as nano sources of heat and wavefront sensing was employed for temperature sensing (Figure 26). This approach encouraged studies based on sub-second time scales. It was revealed that the duration of temperature rise is as relevant as the temperature rise while dealing with distortion of cells induced by heat. With the heating of individual cells, cells can have stressed either in a physiological range of temperature values or distort over 43 °C. A single cell heating produces lower thermal inertia which helps in sudden variation in temperature (around 1 ms). Thus, this technique saves time concerning the amount of sample to be prepared. Moreover, this helps in heating the subcellular region by focusing the laser rather than extending it on the complete area of the cell. Since this technique is label-free, temperature-sensitive fluorescent molecules are not required to tag the cells. It also offers the scope to investigate important queries regarding cellular stress mechanisms. This study triggered a new field of research known as thermal biology at the single-cell level. Future investigations can be performed with respect to the heat-shock reaction of single-cells, while the laser is targeted at the sub-micron scale.

## 4. Limitations and Future Prospects

The primary objective of this review is to discuss various physical methods for cell disruption which are used for different biomolecular delivery at the single-cell level and their analysis. Microfluidic physical approaches for drug delivery are capable of single-cell transfection directly without any carrier vectors. They have various advantages such as easy preparation, transfection of large molecules, and safe manipulation. Ideally, a perfect cell poration technique should display excellent transfection efficiency and low toxicity. Among all the physical methods, electroporation has been considered as one of the most efficient methods for single-cell transfection, both in vitro and in vivo. This is mainly because the electroporation technique can deliver biomolecules in a large number of cells together within a short time period compared to other methods. Moreover, electroporation is capable of transfecting the primary cell types that are non-viral transfection agents. However, the cell viability of the electroporation method is found to be unsatisfactory. With the mechanoporation method, the cell transfection efficiency is less than electroporation and the delivery of molecules into the cell nucleus is not easy. However, mechanoporation depends on the stress created on the cell membrane by the microfluidic channel and does not involve any external forces such as the electric field, magnetic field, ultrasonic wave, and microneedles, etc. However, with the combination of mechanical stress and electric field, high throughput single-cell transfection with high cell viability can be achieved in different cell types, which is promising for single-cell analysis, and in the future, the technique can be used for specific drug resistance testing or personalized medicine purpose. The magnetoporation also has a lesser transfection efficiency than electroporation, but it improves the delivery speed using magnetic transfection reagents. Similarly, Sonoporation displays lower transfection efficiency when compared to electroporation. Due to this, various ultrasound contrast agents are used to enhance the transfection efficiency of Sonoporation. One of the major limitations of Sonoporation is the difficulty in precisely controlling the cell poration. Among all the physical methods, optoporation is promising in enhancing single-cell transfection efficiency and cell visibility. However, the techniques still have low throughput and different cell type delivery is under investigation.

The magnetoporation can be used to deliver difficult-to-transfect cell-like neuronal stem cells. However, the transfection efficiency and cell viability need to improve. In this review, we have discussed various physical methods such as electroporation, photoporation, sonoporation, magnetoporation, and thermoporation to conduct single-cell intracellular delivery and analysis. However, in most cases, lower throughput has been one of the limitations and has to be improved drastically for commercial applications in disease treatment. As we discussed, researchers across the world have been conducting numerous experiments to enhance the throughput and cell viability in the case of single-cell drug delivery systems. Currently, investigations are conducted on combining two different cell poration techniques in a single microfluidic system to enhance the transfection efficiency, cell viability, and to achieve different types of biomolecules delivery into different cell types. This review paper has discussed combining electroporation and mechanoporation as well as combining electroporation and sonoporation which yielded much better results; this indicates that more experiments need to be conducted in this area. In the future, it is expected to develop a microfluidic-based high throughput automated single-cell transfection platform, which will be enabled for personalized medicine, regenerative medicine, cellular therapeutics and diagnostics, and biological and biomedical application purpose.

To summarize, as given in Table 1, every physical method has its advantages and limitations. Thus, it is difficult to rate these methods concerning their transfection efficiencies alone. The decision to apply one method depends on the applications, their characteristics, cell types, and cargo sizes.

## 5. Conclusions

To conclude, single-cell therapy using microfluidic-based physical methods is an emerging field and it has huge potential to be explored in the applications of drug delivery, drug screening, therapeutics, and diagnostics development. For single-cell transfection and analysis, it is important to exert accurate physical energy to disrupt the targeting cell membrane without any damage to the neighboring tissues. The biological and chemical methods have proven much better transfection efficiency than the physical method and this issue must be addressed in the coming years. However, both the above-mentioned methods can perform bulk cell analysis, which can provide average data. The microfluidic-based physical methods are promising and capable of high throughput single-cell transfection with high cell viability, although all of these physical methods based transfections require a breakthrough for clinical applications. Thus, by combining the future technological advancements to disrupt cell membranes, we expect that improvement can be made in the area of catalyzing breakthroughs in delivery applications which could vary from fundamental research to ex vivo cell-based therapies.

## Figures and Tables

**Table 1:** Summary of Physical Membrane Disruption Methods.

Physical Method	Principle	Materials	Advantages	Limitations	References
Thermoporation	Cell membrane disruption by heat transfer.	Laser source to induce heat on cells.	Low heat due to the non-invasive approach.	Low penetration depth; Excess heat can induce thermohaemolysis.	[170,171]
Magnetoporation	Cell membrane disruption by the magnetic field.	Magnetic field; Magnetic transfection agents.	Non-invasive; High efficiency; Allows high selectivity and sensitivity.	Throughput is low; Cell retrieval is not easy.	[47,172]
Sonoporation	Cell membrane disruption by ultrasonic wave.	Ultrasound probe; Ultrasound contrast agents.	High efficiency; Simple fabrication process; Safer compared to the optical method.	Expensive fabrication and calibration process.	[49,173,174,175]
Mechanoporation	Cell membrane disruption by mechanical stress.	Microchannels to create stress on cells.	High throughput; High efficiency; High cell viability.	There could be a trade-off between high cell viability and high transfection efficiency.	[80,83,176,177]
Optoporation	Cell membrane disruption by the laser pulse.	Laser microscope system; Nanorods/Nanoparticles.	Contactless delivery method; High transfection efficiency.	Setup is expensive; Needs quality optical resolution.	[144,145]
Electroporation	Cell membrane disruption by the electric field.	Electrodes; Pulse generator.	Simplicity; Cheaper; Absence of vector; High delivery efficiency.	Low throughput; Only cells responding to the electric field be used.	[178,179,180]
Particle Bombardment	Cell membrane disruption by shooting with biolistic particles.	Metallic particles; High voltage generative devices.	Used in transfecting superficial tissue; High throughput.	Inflammatory response.	[181,182]
Microinjection	Disruption of the cell membrane by micro/nanoneedles.	Microneedles; Syringe pumps.	High throughput; The volume of cargo can be controlled.	Expensive setup; Low efficiency; Skilled technician needed.	[44,63,183,184]

## Data Availability

Not applicable.
